# The progression of initial symptoms and its relationship with the clinical course in adult-onset Still’s disease: from the KEIO-AOSD cohort

**DOI:** 10.1016/j.ero.2025.09.010

**Published:** 2025-10-23

**Authors:** Hiroya Tamai, Jun Kikuchi, Tsutomu Takeuchi, Yuko Kaneko

**Affiliations:** 1 Division of Rheumatology, Department of Internal Medicine, Keio University School of Medicine, Tokyo, Japan.; 2 Saitama Medical University, Iruma, Japan

## Abstract

**Objectives:**

Adult-onset Still’s disease presents with nonspecific and heterogeneous features. This study aims to elucidate the development of initial symptoms and their relationship with the disease course in adult-onset Still’s disease.

**Methods:**

We retrospectively reviewed consecutive patients with adult-onset Still’s disease who had been enrolled in the KEIO-AOSD cohort from April 2012 to July 2024. Clinical information was collected from their medical records. Detailed initial symptoms and their relationship with disease courses were analyzed.

**Results:**

A total of 89 patients were included. The mean age at diagnosis was 46.3 ± 18.5 years, and 73.0% were females. The median time from the onset of initial symptoms to the first clinic visit, the first blood testing, satisfaction of Yamaguchi’s criteria, and treatment initiation were 5.0, 10.0, 21.0, and 35.0 days, respectively. As initial symptoms, fever, was observed in 50.6%, rash and arthralgia in 37.1% each, and sore throat in 30.3%. Fever and sore throat rapidly increased within 2 weeks prior to Yamaguchi’s criteria satisfaction. Patients with rash as an initial symptom tended to experience a delay in taking blood testing and satisfying Yamaguchi's criteria. Patients presenting with a sore throat as an initial symptom tended to show higher ferritin levels and experience macrophage activation syndrome. No significant differences were observed in long-term outcomes.

**Conclusions:**

Early clinical courses of adult-onset Still’s disease varied depending on initial symptoms. Focusing on initial symptoms in clinical practice can contribute to earlier diagnosis and better management.

**WHAT IS ALREADY KNOWN ON THIS TOPIC**

• Adult-onset Still’s disease presents with nonspecific features and heterogeneous presentations, often resulting in delayed diagnosis and suboptimal therapeutic management.
• Early treatment initiation during the “window of opportunity” is considered important to minimise damage accrual and potentially reduce the risk of life-threatening complications such as macrophage activation syndrome.

**WHAT THIS STUDY ADDS**

• This study revealed distinct temporal patterns of individual symptom prior to diagnosis: the frequency of fever and sore throat increased rapidly within the 2 weeks preceding diagnosis.
• Furthermore, the clinical course differed depending on the initial presenting symptom: patients presented with skin rash tended to experience delayed diagnosis, whereas those presented with sore throat experienced higher serum ferritin levels and a higher incidence of macrophage activation syndrome.

**HOW THIS STUDY MIGHT AFFECT RESEARCH, PRACTICE, OR POLICY**

• This study highlights the variability in time to diagnosis and subsequent clinical course based on the initial presenting symptom, suggesting that focusing on the initial symptom may contribute to earlier diagnosis, effective risk stratification, and optimized clinical management.

Alt-text: Unlabelled box

## INTRODUCTION

Still’s disease, a combined disease spectrum of systemic juvenile idiopathic arthritis and adult-onset Still’s disease, is an autoinflammatory disease characterised by severe systemic symptoms and laboratory findings such as spiking fever, skin rash, arthritis, leucocytosis, liver dysfunction, and high serum ferritin levels [[1], [2], [3]]. Whereas early treatment initiation during the “window of opportunity” is recommended to minimize damage accrual [4], these initial nonspecific features and heterogeneous presentations frequently lead to delayed and suboptimal management of patients, potentially increasing the risk of life-threatening complications such as macrophage activation syndrome [1].

Adult-onset Still's disease is traditionally categorised into 3 subtypes: monocyclic, polycyclic, and chronic articular types [5], and more recent sophisticated approaches, such as clustering methods, have classified the disease into more detailed clinical subtypes with clinical courses [6,7]. However, in the early stages of the disease, when not all symptoms and signs are presented, how those symptoms manifest and how those symptoms affect clinical courses are not fully undestood. Also, it remains unclear whether initial symptoms at onset make a difference in the subsequent clinical courses that are affected by treatment with biological agents, although IL-1 inhibitors had not been approved for AOSD until recently in Japan [8].

This study aimed to elucidate the patterns of symptom and sign presentation in the early stages of adult-onset Still’s disease and to clarify whether those initial symptoms affect the time courses of diagnosis and management.

## METHODS

### Patients and data collection

We reviewed consecutive patients with adult-onset Still’s disease from the KEIO-AOSD (Keio University – adult-onset Still’s disease) cohort who were diagnosed based on Yamaguchi’s criteria [3] and visited Keio University Hospital from April 2012 to June 2024. To minimize recall bias, we included only cases in which the progression of initial symptoms was thoroughly documented in the medical records based on detailed clinical interviews. Our study was approved by the ethics committee of Keio University School of Medicine, Tokyo, Japan (approval number: 20130506). The 2008 Declaration of Helsinki and the 2008 Ethical Guidelines for Clinical Research by the Japanese Ministry of Health, Labour and Welfare were observed.

### Assessment and definitions

The initial symptom or sign was defined as the earliest manifestation among the 8 components of Yamaguchi’s criteria, as documented in the medical records, including the clinical history obtained through detailed interviews. Fever was assessed based on recorded body temperatures, which included both those measured in the hospitals and reported by patients. Rash was considered present based on the judgement of rheumatologists as characteristic of adult-onset Still’s disease, which also included both physical examinations and patient-reported symptoms. The date of satisfaction of Yamaguchi’s criteria was defined as the date on which at least 5 criteria, including at least 2 major criteria, were met, irrelevant of the exclusion of other differential diagnoses. The treatment initiation was the date of the initiation of glucocorticoid or an immunosuppressive drug without glucocorticoids [9]. Inadequate response to the initial treatment was defined as persistent disease activity requiring an increase in the glucocorticoid dose or an addition of intravenous methylprednisolone pulse therapy within 90 days from the initial treatment [9]. Recurrence was defined as an exacerbation in disease activity after resolution of symptoms associated with Still’s disease, requiring treatment intensification with either an increase in glucocorticoid dose by at least 50% or the initiation of new immunosuppressive drugs and/or a biological agent targeting inflammatory cytokines [8,9]. Macrophage activation syndrome was diagnosed according to the criteria for systemic juvenile idiopathic arthritis proposed by the European Alliance of Associations for Rheumatology (EULAR)/American College of Rheumatology/Paediatric Rheumatology International Trials Organisation [10].

### Statistical analysis

Parametric continuous values are presented as the mean with SD, and nonparametric continuous values are presented as the median with IQR (25%-75%). Two continuous variables were compared using Student’s *t* test for parametric variables or the Wilcoxon signed-rank test for nonparametric variables. Three or more continuous variables were compared using analysis of variance for parametric variables or the Kruskal-Wallis test for nonparametric variables. For multiple comparisons of continuous variables among more than 2 groups, Tukey’s test for parametric variables and the Steel-Dwass test for nonparametric variables were applied to account for multiplicity. Categorical variables were compared using Pearson’s chi-square test or Fisher’s exact test. For multiple comparisons of categorical variables among more than 2 groups, the Holm-Bonferroni method was applied to adjust for multiplicity. Survival rates of the 2 groups were compared with the log-rank test. Weighted analyses based on the number of initial symptoms were conducted for sensitivity analysis. Imputation for missing data was not conducted. All statistical analyses were performed using R version 4.3 (R Foundation for Statistical Computing, Vienna, Austria). A *P* value < .05 was considered statistically significant for superiority hypotheses.

## RESULTS

### Baseline characteristics and time course until treatment initiation

A total of 89 patients with newly diagnosed adult-onset Still’s disease, according to Yamaguchi’s criteria, with sufficient information, were included in this study. Patient demographics and characteristics at diagnosis are shown in Supplementary Table S1. The mean age at diagnosis was 46.3 ± 18.5 (SD) years, and 65 (73.0%) of them were female. Fever was observed in 88 (98.9%) patients, skin rash in 80 (89.9%), arthralgia/arthritis in 79 (88.8%), sore throat in 60 (67.4%), lymphadenopathy and/or splenomegaly in 67 (75.3%), leucocytosis in 73 (82.0%), liver dysfunction in 76 (85.4%), and negative rheumatoid factor and antinuclear antibody in 57 of 88 (64.8%). The mean CRP level was 15.8 ± 8.7 mg/dL, and the median serum ferritin level was 4412 (IQR 1438, 8735) ng/mL.

### Periods from the symptom onset to clinic visit, blood tests, criteria satisfaction, and treatment initiation

Supplementary Figure S1 shows the median periods of time from disease symptom onset to treatment initiation. The median days from disease symptom onset to the first clinic visit was 5.0 days, to the first blood test was 10.0 days, to the satisfaction of Yamaguchi’s criteria, except for exclusion of other differential diagnosis, was 21.0 days, and to treatment initiation was 35.0 days.

### Manifestation of each symptom and sign

**Figure 1:**
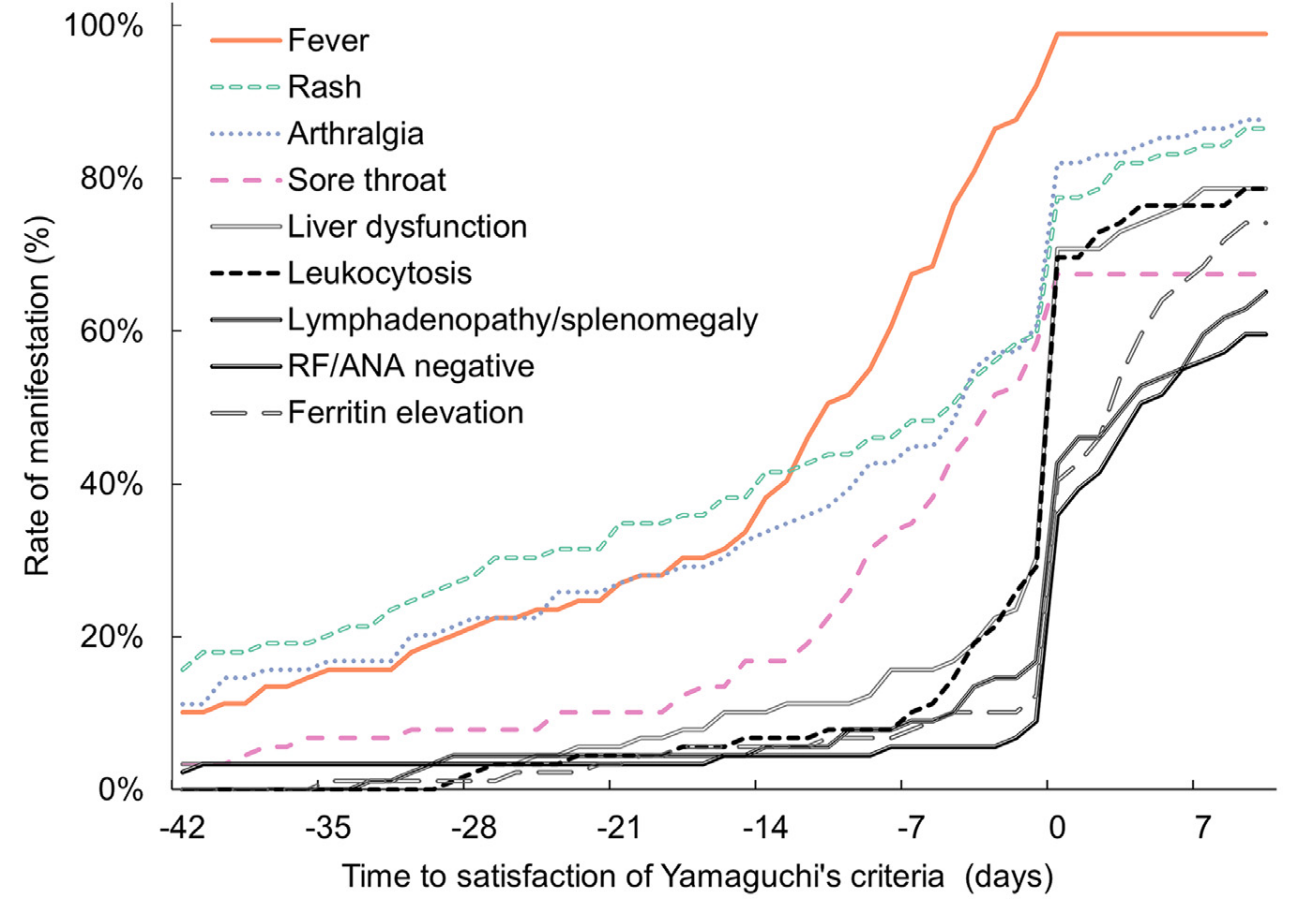
Cumulative incidence of individual symptoms and signs. The cumulative incidence of individual symptoms and signs is shown as the number of days to the satisfaction of Yamaguchi's criteria. Rashes and arthralgia gradually increased over time, while fever and sore throat rapidly increased from 2 weeks prior to the satisfaction of Yamaguchi’s criteria. ANA, antinuclear antibody; RF, rheumatoid factor.

The cumulative incidences of individual symptoms and signs are shown in Figure 1, presented as the time to the satisfaction of Yamaguchi's criteria. Rashes, arthralgia, and fever were the main symptoms which appeared almost 6 weeks before the satisfaction of Yamaguchi's criteria. Rashes and arthralgia gradually increased over time, while fever and sore throat rapidly increased from 2 weeks prior to the satisfaction of Yamaguchi’s criteria. Most laboratory and physical/imaging findings increased just before the satisfaction of Yamaguchi’s criteria, likely because these findings led to the fulfilment of Yamaguchi’s criteria.

### Ferritin measurement and its significance in the satisfaction of Yamaguchi’s criteria

Ferritin levels were measured in only 23 of 88 patients (26.1%) before satisfying Yamaguchi’s criteria. The median period from symptom onset to ferritin measurements was 18 (11, 37) days. Adding ferritin elevation (>200 ng/mL or >1000 ng/mL) as a minor criterion to Yamaguchi’s criteria would have little effect on shortening the time until satisfaction of 5 criteria, including 2 major criteria (Supplementary Fig S2).

### Initial symptoms

**Figure 2:**
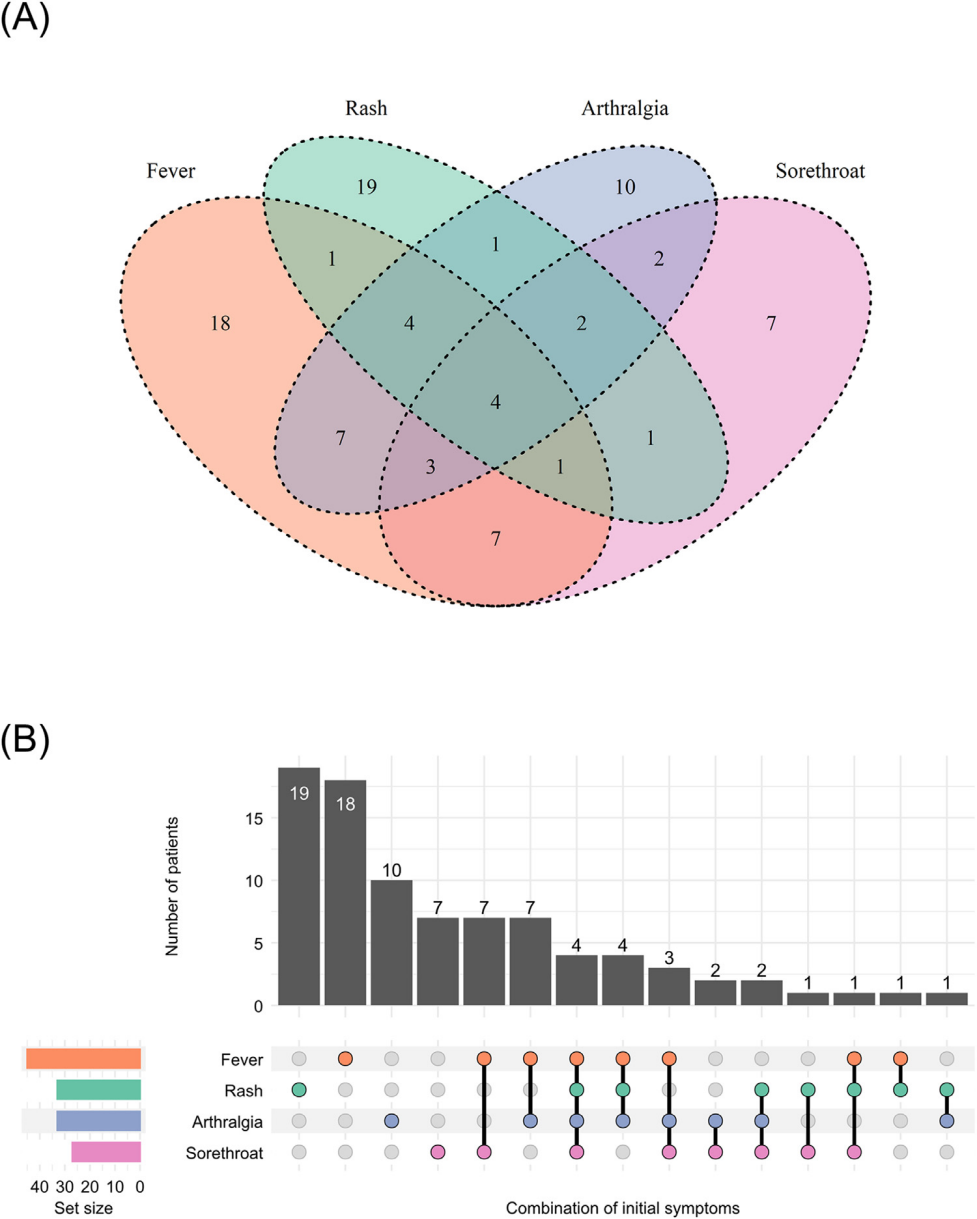
The number of patients with initial symptoms. A, Venn diagram. B, Upset plot. Numbers indicated in the (A) Venn diagram are the number of patients with each combination of symptoms. In (B), the Upset plot, the number of patients with each combination of initial symptoms is represented as bar graphs, ordered from left to right by frequency. Rash tended to appear as an initial symptom without accompanying other symptoms.

Initially, patients (*n* = 89) presented with a total of 143 symptoms or findings, as shown in Supplementary Table S1. We focused on 4 main initial symptoms (fever *n* = 45; rash, *n* = 33; arthralgia, *n* = 33; sore throat, *n* = 27) since only 5 patients presented with initial symptoms or signs other than these 4 manifestations. The combination of those 4 symptoms is shown in a Venn diagram (Fig 2A) and an Upset plot (Fig 2B). Fifty-four patients (62.1%) had only 1 initial symptom, 19 (21.8%) had 2 symptoms, 10 (11.5%) had 3 symptoms, and 4 (4.6%) had 4 symptoms at symptom onset. Patients who had rash as their first symptom were less likely to have the other symptoms at onset (patients with only 1 symptom at onset: fever, 18/45 [40.0%]; rash, 19/33 [57.6%]; arthralgia/arthritis, 10/33 [30.3%]; sore throat, 7/27 [25.9%]; *P* = .05).

### The relationship between the initial symptoms and subsequent clinical manifestations and diagnosis

**Figure 3:**
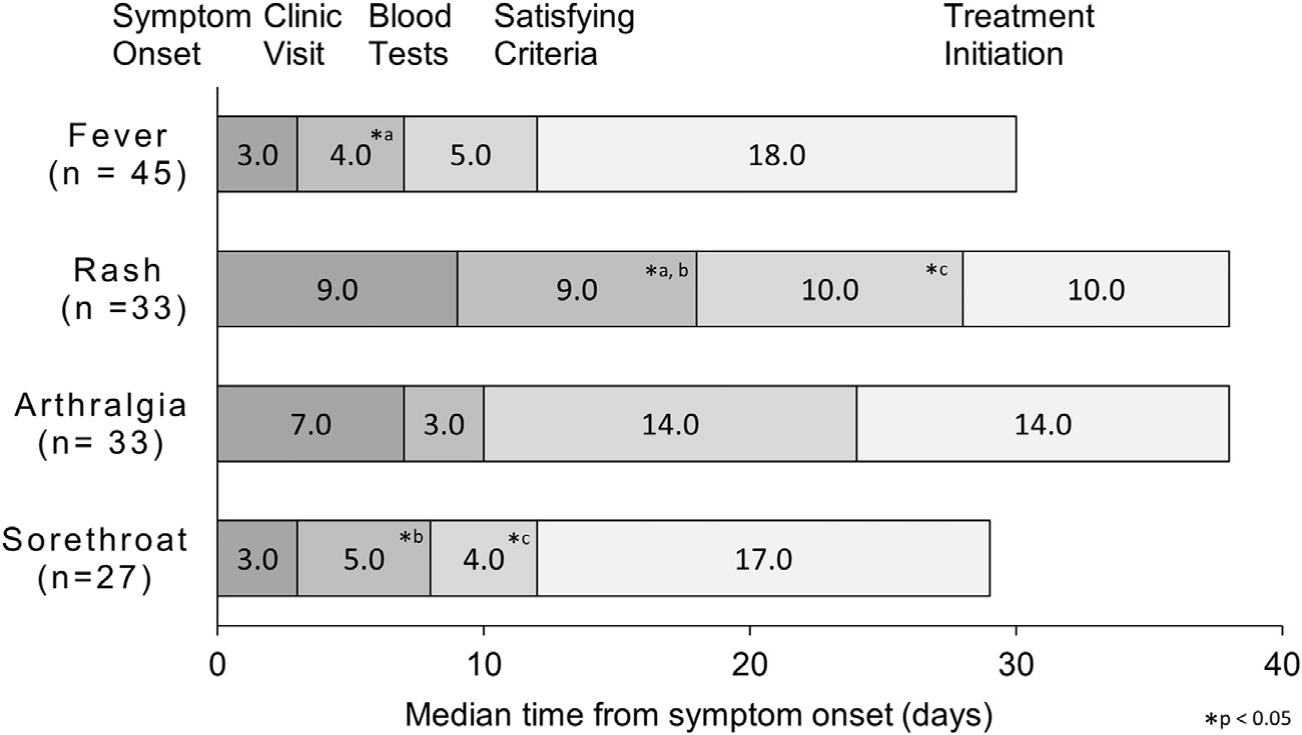
Periods from symptom onset to clinic visit, blood test, satisfying criteria, and treatment initiation by each initial symptom. Combinations marked with asterisks and letters indicate those with *P* values < .05 in the Steel-Dwass test, adjusted for multiple comparisons. Patients with rash as the initial symptom took a longer time from disease onset to medical facility visit and blood test, and satisfaction of Yamaguchi’s criteria compared to those with fever or sore throat, although the median days from the onset to treatment initiation were not significantly different in the 4 groups.

We stratified patients according to the 4 main initial symptoms described above and compared the subsequent time courses and clinical characteristics (Fig 3, Table).

**Table:** Differences in patient demographics and disease characteristics by initial symptoms

	Fever (*n* = 45)	Rash (*n* = 33)	Arthralgia (*n* = 33)	Sore throat (*n* = 27)	*P*
Patients’ characteristics					
Age, y	41.4 (18.7)	45.9 (16.7)	46.1 (18.6)	47.7 (16.8)	0.464
Female, *n* (%)	30 (66.7)	29 (87.9)	23 (69.7)	16 (59.3)	0.063
Incidence of Yamaguchi’s criteria, *n* (%)					
Fever	45 (100)	32 (97.0)	32 (97.0)	26 (96.3)	0.546
Rash	40 (88.9)	33 (100)	26 (78.8)	24 (88.9)	0.030*
Arthralgia/arthritis	38 (84.4)	31 (93.9)	33 (100)	21 (77.8)	0.015*
Sore throat	29 (64.4)	25 (75.8)	17 (51.5)	27 (100)	< 0.001*
Lymphadenopathy and/or splenomegaly	35 (77.8)	24 (72.7)	25 (75.8)	22 (81.5)	0.897
Leucocytosis	37 (82.2)	29 (87.9)	25 (75.8)	21 (77.8)	0.595
Liver dysfunction	39 (86.7)	25 (75.8)	26 (78.8)	23 (85.2)	0.582
Negative rheumatoid factor and antinuclear antibody	29 (64.4)	21/32 (65.6)	21 (63.6)	21 (77.8)	0.636
Laboratory data before treatment initiation					
Neutrophil count, maximum, × 10^3^/μL	14,198 (5264) (*n* = 37)	14,761 (5848) (*n* = 27)	13,982 (5900) (*n* = 28)	13,915 (5668) (*n* = 24)	0.946
Haemoglobin, minimum, g/dL	10.5 (1.6) (*n* = 43)	10.6 (1.5) (*n* = 30)	10.5 (1.7) (*n* = 31)	10.8 (1.6) (*n* = 25)	0.915
Platelet count, maximum, × 10^4^/μL	34.6 (13.7) (*n* = 44)	34.5 (13.7) (*n* = 31)	39.5 (18.9) (*n* = 32)	35.1 (16.8) (*n* = 26)	0.517
Lactate dehydrogenase, maximum, U/L	566 (364, 796) (*n* = 43)	495 (386, 751) (*n* = 31)	465 (265, 579) (*n* = 32)	572 (476, 852) (*n* = 26)	0.085
C-reactive protein, maximum, mg/dL	17.2 (8.3) (*n* = 44)	14.7 (9.2)	15.7 (8.2)	15.5 (8.3)	0.606
Erythrocyte sedimentation rate, mm/h	97 (28) (*n* = 32)	88 (29) (*n* = 22)	100 (20) (*n* = 24)	84 (36) (*n* = 20)	0.184
Soluble IL-2 receptor, maximum, U/mL	1405 (851, 1693) (*n* = 30)	1220 (827, 1470) (*n* = 19)	1240 (64, 1495) (*n* = 19)	1605 (1392, 2389) (*n* = 20)	0.150
Matrix metalloproteinase-3, maximum, ng/mL	80 [42, 260] (*n* = 30)	94 [58, 140] (*n* = 18)	127 [76, 271] (*n* = 21)	85 [55, 123] (*n* = 20)	0.354

Data are *n* (%), mean (SD), and median [Q1-Q3]. *P* values were results of Fisher’s exact test for categorical variables, analysis of variance for parametric continuous variables, or the Kruskal-Wallis test for nonparametric continuous variables among the 4 groups. Asterisks (*) indicate *P* value < .05. Patients with more than one initial symptom were included in multiple groups. The results adjusted for the number of initial symptoms are presented in Supplementary Table S3.

Patients with rash as an initial symptom tended to experience delays in clinic visits (fever, 3 [1,8] days; rash, 9 [3, 21] days; arthralgia, 7 [2, 21]; sore throat, 3 [1,9] days; *P* = 0.074, rash vs fever) and blood testing (fever, 7 [3,15] days; rash, 18 [9, 32] days; arthralgia, 10 [5, 21]; sore throat, 8 [4,15] days; *P* = .005, rash vs fever; *P* = .044, rash vs sore throat), leading to a longer time to satisfy Yamaguchi’s criteria (fever, 12 [7, 29] days; rash, 28 [14, 42] days; arthralgia, 24 [9, 47]; sore throat, 12 [7, 24] days; *P* = .056, rash vs fever; *P* = .040, rash vs sore throat, Fig 3). The median days from the symptom onset to treatment initiation were not significantly different among the 4 groups (*P* = .291).

Whereas more than 60% of patients had only 1 symptom at onset, the number of symptoms increased in all patients and there was no big difference at the time of satisfaction of Yamaguchi’s criteria, except for patients presented with sore throat having less other symptoms (*P* = .001, vs fever; *P* = .025, vs rash; *P* < .001, vs arthralgia, Table).

**Figure 4:**
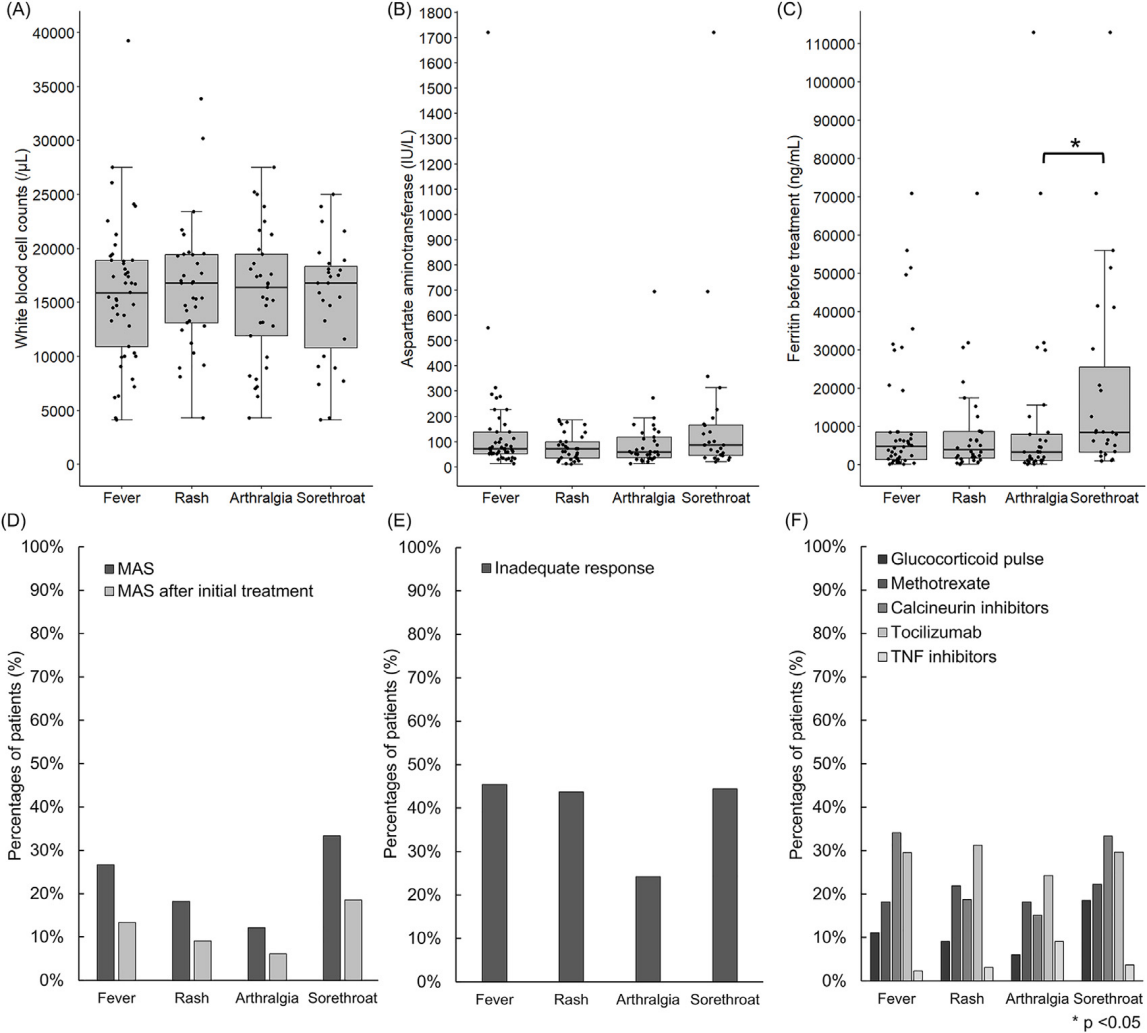
Comparison of baseline findings and treatment courses by initial symptoms. Comparison of (A) white blood cell counts, (B) aspartate aminotransferase levels, and (C) ferritin levels, (D) proportion of macrophage activation syndrome, (E) proportion of inadequate response to initial treatment, and (F) treatment within 90 days among 4 groups based on initial symptoms. The asterisks (*) indicate between-group comparisons with *P* values < .05 in tests adjusted for multiple comparisons. Patients presented with a sore throat showed higher serum ferritin levels before treatment initiation. MAS, macrophage activation syndrome; TNF, tumor necrosis factor.

As for characteristics, patients presented with sore throat predominantly to be female (87.9%, Table) and showed higher serum ferritin levels before treatment initiation (fever, 4796 [1400, 8539] ng/mL; arthralgia/arthritis, 3293 [1119, 7951] ng/mL; rash, 3935 [1733, 8703] ng/ml; sore throat, 8388 [3351, 25492]; *P* = .048, sore throat vs arthralgia, Fig 4C). They also tended to be complicated with macrophage activation syndrome, although not statistically significant (fever, 26.7%; rash, 18.2%; arthralgia, 12.1%; sore throat, 33.3%; *P* = 0.063, arthralgia vs sore throat, Fig 4D).

### Treatment pattern by initial symptoms

Among 89 patients, 85 patients were treated with glucocorticoids, with the mean initial dose of prednisolone 43.5 ± 19.1 mg/d (Supplementary Table S2). Two patients did not receive glucocorticoids, biological agents, or immunosuppressive drugs, and the other 2 patients were started on biological agents without glucocorticoids. Nine patients (10.1%) received glucocorticoid pulse therapy. There was no difference in initial prednisolone dosage among patients with each initial symptom (fever, 44.5 ± 19.4 mg/d; rash, 42.1 ± 20.8 mg/d; arthralgia, 40.5 ± 14.5 mg/d; sore throat, 46.6 ± 21.4 mg/d; *P* = .628). Within 90 days from the initial treatment, 50 patients (57.5%) started biological agents or immunosuppressive drugs in addition to glucocorticoids, most frequently with tocilizumab (29.9%), followed by cyclosporin (20.7%) and methotrexate (17.2%, Supplementary Table S2). Patients presented with fever tended to be treated with calcineurin inhibitors (fever, 34.1%; rash, 18.8%; arthralgia, 15.2%; sore throat, 33.3%; *P* = .071, fever vs arthralgia, Fig 4F).

Forty patients (46.0%) had inadequate response to initial treatment and needed an increase in glucocorticoid dose at a median period of 14 (8,21) days from initial treatment. Patients presented with arthralgia tended to have lower rates of inadequate response to initial treatment, although not statistically significant (fever, 45.5%; rash, 43.8%; arthralgia, 24.2%; sore throat, 44.4%; *P* = .062, fever vs arthralgia, Fig 4E). A total of 10 cases of macrophage activation syndrome occurred after treatment initiation, with a median time of 29 (16, 163) days from treatment initiation.

### Long-term outcomes by initial symptoms

During the median observation period of 7.8 (3.6, 10.6) years, 40 patients (44.9%) experienced recurrence after 1.4 (0.6, 2.9) years from the initial treatment. On the other hand, 53 (62.4%) patients succeeded in glucocorticoid discontinuation after 2.5 (1.3, 4.7) years. The incidence of recurrence or glucocorticoid discontinuation was not related to initial symptoms (*P* = .86, *P* = .87, respectively, Supplementary Figures S3 and S4).

### Sensitivity analysis

In the comparison of early disease courses and long-term outcomes based on initial symptoms, we conducted sensitivity analysis by adjusting the number of initial symptoms in each patient. Similar trends were observed about the time course from the onset, baseline characteristics, and long-term outcomes (Supplementary Figs S5-S7 and Supplementary Tables S3 and S4).

## DISCUSSION

Our study demonstrated that initial symptoms were various in patients with adult-onset Still’s disease and were related to the delay in diagnosis and subsequent clinical courses. Rash as the initial symptom tended to be present alone and was associated with a longer period to satisfy Yamaguchi's criteria, and sore throat tended to be related to remarkably increased ferritin levels and macrophage activation syndrome. Initial symptoms were not related to long-term outcomes.

In our study, the frequency of macrophage activation syndrome and the proportion of inadequate response to initial treatment tended to differ by initial symptoms. Although the relationship between sore throat and macrophage activation syndrome in previous studies was conflicting [[11], [12], [13]], our study revealed that macrophage activation syndrome tended to be more frequent in patients who presented with sore throats as the initial symptoms compared to those with arthralgia. Our study also showed that patients with arthralgia as the initial symptom had a lower proportion of inadequate response to initial treatment. Whereas clinical courses of adult-onset Still’s disease are classically categorised into 3: monocyclic systemic, polycyclic systemic, and chronic [5], the recent widespread use of biological agents such as IL-1 inhibitors and an IL-6 inhibitor has decreased the number of patients with a chronic articular course [14]. Our study suggests that focusing on initial symptoms, which may reflect differences in the underlying disease pathophysiology, is useful to predict subsequent clinical courses.

In our study, the median period from initial symptoms to satisfaction of Yamaguchi’s criteria was 3 weeks, and the time to treatment initiation was 5 weeks. Especially, patients with rash as an initial symptom had a longer time to satisfy Yamaguchi’s criteria. It is partly not only due to the natural time course of developing several symptoms and signs but also due to little suspicion of Still’s disease among primary physicians, as most patients were treated with several antibiotics without any infectious sites or being checked for ferritin levels (data not shown). Although rash is known to be characterised by concomitant appearance with fever, a considerable number of patients manifested rash without accompanying fever at disease onset. Also, a study reported that skin eruptions other than salmon pink rashes with fever can lead to diagnostic delays [15]. In Germany, the implementation of clinical guidelines has reduced the time to diagnosis [16], highlighting the importance of educating general internists and dermatologists, as EULAR/PReS recommendations stated [1].

The current study showed that adding ferritin levels as a minor criterion had little impact on reducing the time to diagnosis. Yamaguchi et al mentioned in their development of Yamaguchi’s criteria that ferritin levels were not included as a criterion due to their limited contribution to improving sensitivity and specificity [3]. Actually, in our study, at the time of the first ferritin measurement, three-quarters of patients had already met Yamaguchi’s criteria. However, it also suggests that those measurements may have been too late for the diagnostic process. If adult-onset Still’s disease had been suspected earlier and ferritin had been measured sooner, its contribution to reducing the time to diagnosis might have improved. Furthermore, the identification of more specific biomarkers is warranted, as they may facilitate earlier diagnosis. Future studies are needed to investigate the possibility.

The strength of this study is that patients were categorized according to their initial symptoms based on medical records containing detailed patient history from comprehensive clinical interviews. Recently, various subtypes of adult-onset Still’s disease have been proposed; however, these classifications are usually based on information available at the time of diagnosis or clinical course after treatment initiation [6,7,[17], [18], [19]]. Focusing on initial symptoms allows for a clear and consistent classification, which may be beneficial in clinical practice.

This study also has some limitations. First, the study was retrospective with a limited number of patients due to the rarity of adult-onset Still’s disease. Second, the collected initial symptoms were based on patient self-reports, which may lead to some recall bias. However, we believe that referring to medical records containing detailed interviews conducted at the time of clinical care allowed us to obtain accurate information while minimizing recall bias. Third, although Yamaguchi’s criteria specify duration of fever (≥1 week) and arthralgia (≥2 weeks), it was difficult to assess the exact duration of symptoms, which may have resulted in a possible discrepancy in actual dates of achieving Yamaguchi’s criteria. Despite these limitations, we believe that classifying adult-onset Still’s disease based on initial symptoms enables clinically meaningful stratification related to subsequent disease course.

In conclusion, early clinical courses of adult-onset Still’s disease differ depending on initial symptoms. Focusing on initial symptoms can contribute to an earlier diagnosis and better management.

## CRediT authorship contribution statement

**Hiroya Tamai:** Writing – review & editing, Writing – original draft, Visualization, Project administration, Methodology, Formal analysis, Data curation, Conceptualization. **Jun Kikuchi:** Writing – review & editing, Conceptualization. **Tsutomu Takeuchi:** Writing – review & editing, Supervision. **Yuko Kaneko:** Writing – review & editing, Writing – original draft, Supervision, Project administration, Conceptualization.

## Competing interests

H.T. received honoraria from AbbVie, Asahi Kasei, Eisai, and Novartis; and support for attending meetings from PhRMA. J.K. received speaker fees from AbbVie, Asahi Kasei, AstraZeneca, Chugai, Eisai, Eli Lilly, Glaxo Smith Kline, Janssen, Kissei, Mitsubishi-Tanabe, Taisho, and UCB. T.T. received consulting fees from AbbVie, Eli Lilly, Gilead Sciences, Mitsubishi-Tanabe, and Taisho; and honoraria from AbbVie, Astellas, AstraZeneca, Chugai, Eisai, Eli Lilly, Gilead Sciences, Janssen, Mitsubishi-Tanabe, Pfizer, and Taisho. Y.K. received grants from AbbVie, Asahi Kasei, Ayumi, Boehringer Ingelheim, Chugai, Eisai, Gilead Sciences, Mitsubishi-Tanabe, Taisho, and UCB; consulting fees from AbbVie, Asahi Kasei, Bristol-Myers Squibb, Eli Lilly, Gilead Sciences, Pfizer, Taisho, and UCB; and honoraria from AbbVie, Asahi Kasei, Astellas, AstraZeneca, Ayumi, Bristol-Myers Squibb, Chugai, Daiichi-Sankyo, Eisai, Eli Lilly, Gilead, Glaxo SmithKline, Janssen, Mitsubishi-Tanabe, Novartis, Pfizer, Sanofi, Taisho, and UCB.

## Appendix Supplementary materials

Image, application 1

## Footnotes

Supplementary material associated with this article can be found in the online version at doi:10.1016/j.ero.2025.09.010.
