# Supplementary material for: The progression of initial symptoms and its relationship with the clinical course in adult-onset Still’s disease: from the KEIO-AOSD cohort
Source: EULAR Rheumatol Open. 2025 Oct 23;1(4):331–8. doi: 10.1016/j.ero.2025.09.010 (PMC13292144; doi:10.1016/j.ero.2025.09.010)
Supplement: Supplementary file 1 [file mmc1.pdf]

Supplemental Materials

Contents

Supplementary Figure S1. Periods from symptom onset to clinic visit, blood tests, satisfying criteria, and treatment initiation. 2

Supplementary Figure S2. Percentage of satisfying five components of Yamaguchi's criteria. 3

Supplementary Figure S3. Kaplan-Meier curve for recurrence compared by initial symptoms. 4

Supplementary Figure S4. Kaplan-Meier curve for glucocorticoid discontinuation compared by initial symptoms. 5

Supplementary Figure S5. Periods from symptom onset to clinic visit, blood tests, satisfying criteria, and treatment initiation weighted by the number of initial symptoms. 6

Supplementary Figure S6. Kaplan-Meier curve for recurrence compared by initial symptoms weighted by the number of initial symptoms. 7

Supplementary Figure S7. Kaplan-Meier curve for glucocorticoid discontinuation compared by initial symptoms weighted by the number of initial symptoms. 8

Supplementary Table S1. Patient demographics and disease characteristics. 9

Supplementary Table S2. Treatment and Outcomes 11

Supplementary Table S3. Comparison of demographics and disease characteristics at diagnosis based on initial symptoms adjusted by their number 13

Supplementary Table S4. Comparison of Treatment and Outcomes based on Initial Symptoms Adjusted by their Number 15

Supplementary Figure S1. Periods from symptom onset to clinic visit, blood tests, satisfying criteria, and treatment initiation.

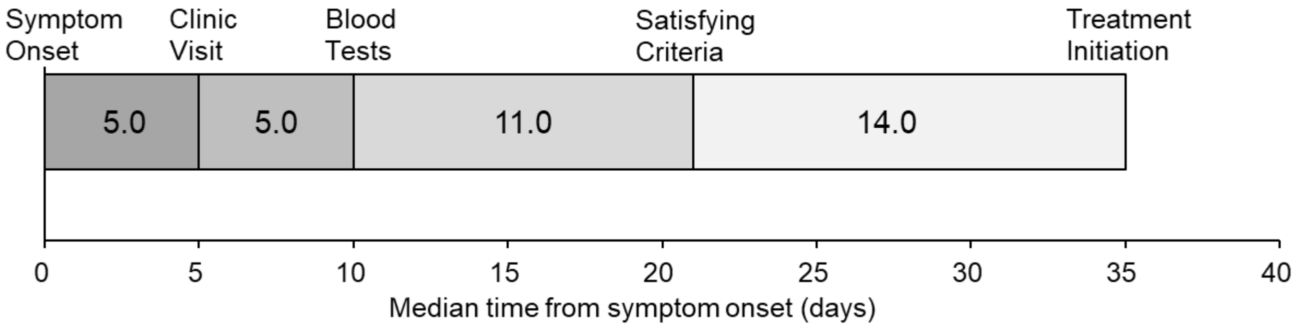

The median time from disease onset to first medical facility visit was 5.0 days, time to the first blood test was 10.0 days, time to satisfaction of Yamaguchi's criteria was 21.0 days, and time to treatment initiation was 35.0 days, with the longest time after satisfaction of Yamaguchi’s criteria until treatment initiation.

Supplementary Figure S2. Percentage of satisfying five components of Yamaguchi's criteria.

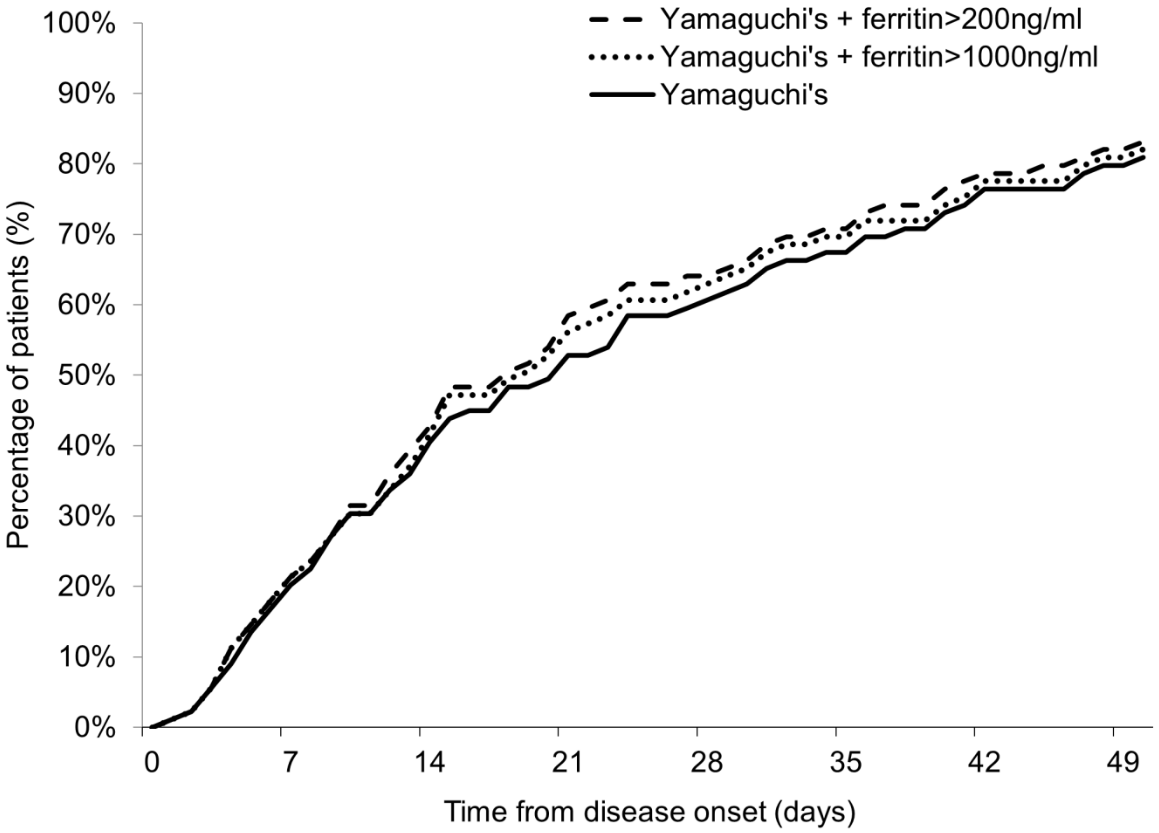

Adding ferritin elevation (>200 ng/ml, or >1000 ng/ml) as a component of the Yamaguchi's criteria had little effect on the satisfaction of five components of the criteria.

Supplementary Figure S3. Kaplan-Meier curve for recurrence compared by initial symptoms.

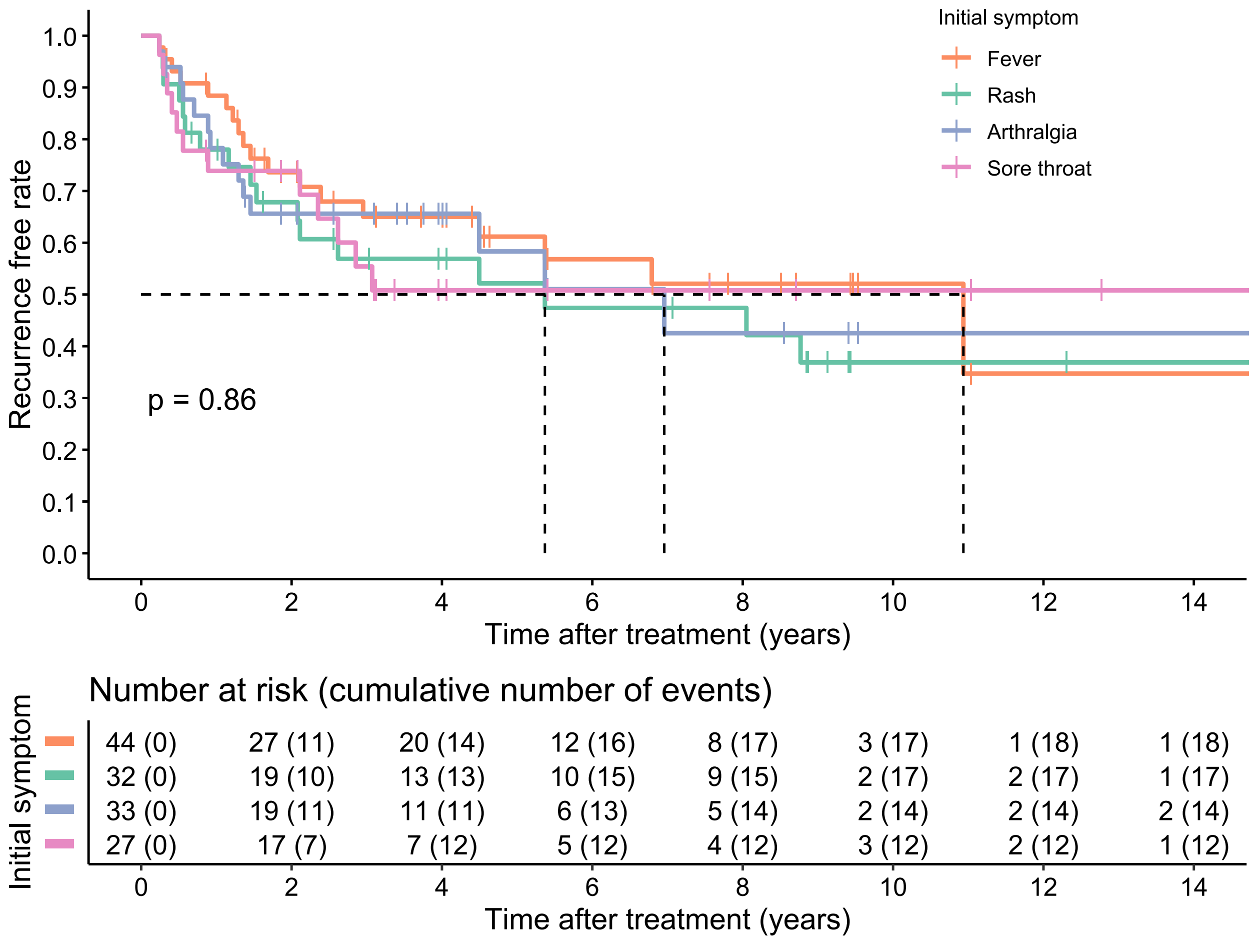

Patients with different initial symptoms didn’t show difference in the rate of recurrence.

Supplementary Figure S4. Kaplan-Meier curve for glucocorticoid discontinuation compared by initial symptoms.

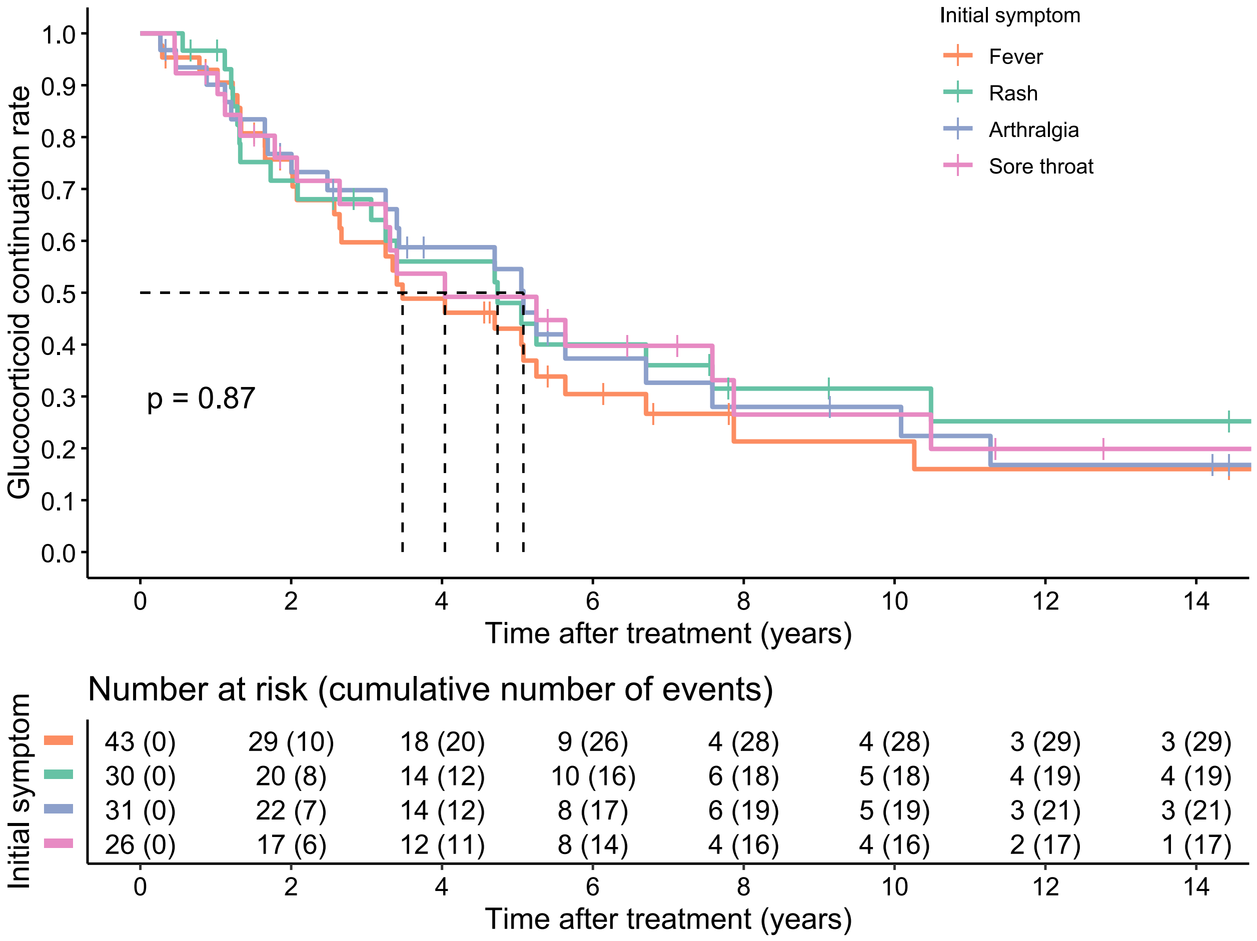

Patients with different initial symptoms didn’t show difference in the rate of glucocorticoid discontinuation.

Supplementary Figure S5. Periods from symptom onset to clinic visit, blood tests, satisfying criteria, and treatment initiation weighted by the number of initial symptoms.

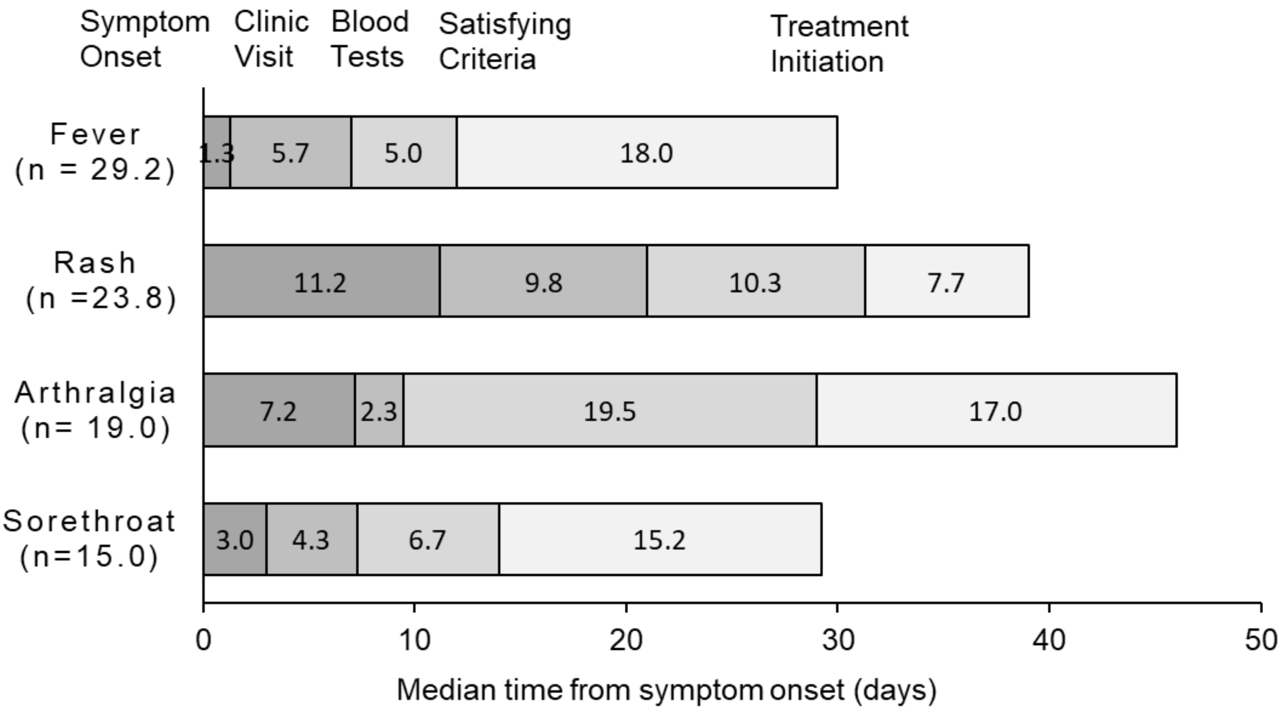

Similar trends were observed about the time course from the onset.

Supplementary Figure S6. Kaplan-Meier curve for recurrence compared by initial symptoms weighted by the number of initial symptoms.

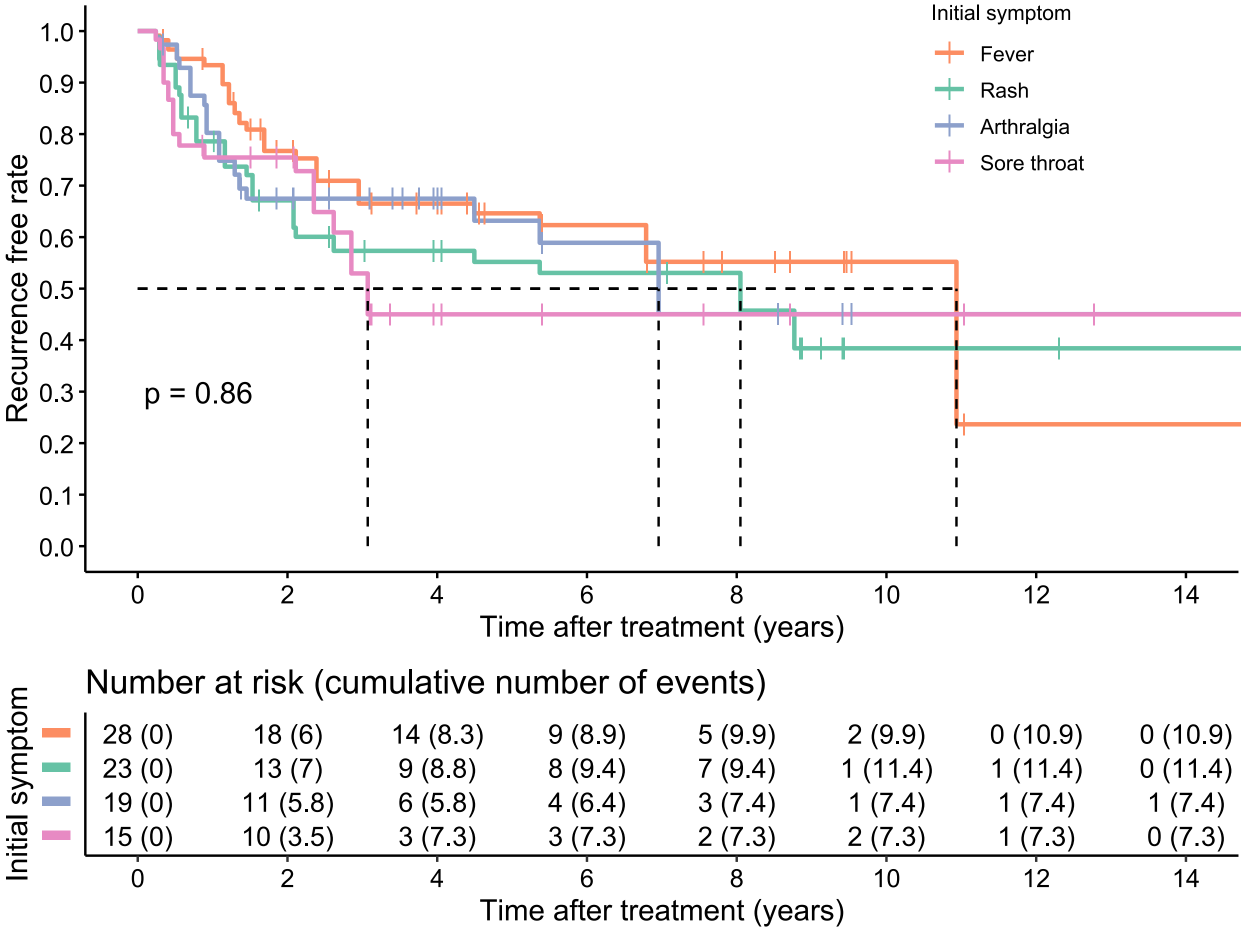

Similar trends were observed about the recurrence.

Supplementary Figure S7. Kaplan-Meier curve for glucocorticoid discontinuation compared by initial symptoms weighted by the number of initial symptoms.

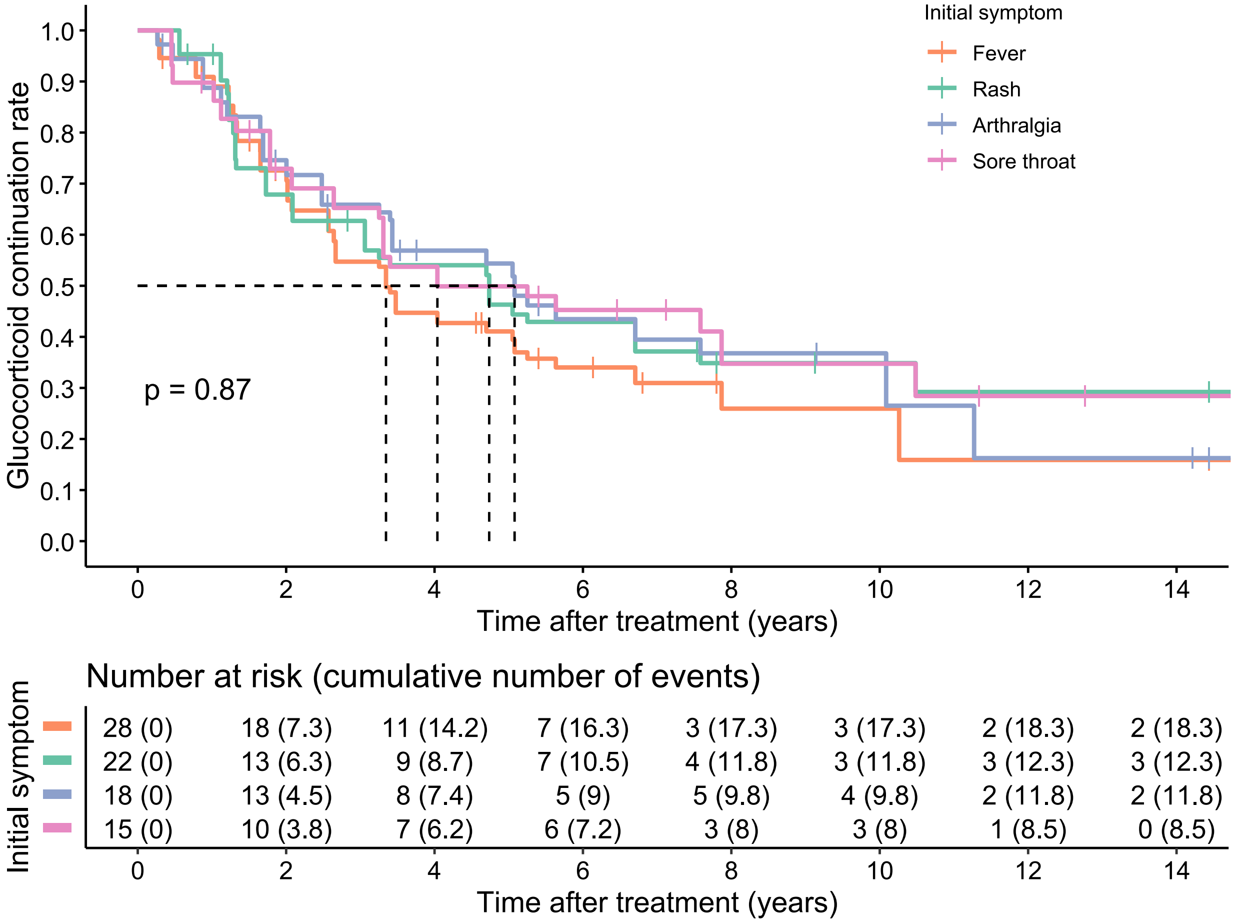

Similar trends were observed about the glucocorticoid discontinuation.

Supplementary Table S1. Patient demographics and disease characteristics.

	All (n = 89)
**Patients' characteristics**	
Age, years	46.3 (18.5)
Female, n (%)	65 (73.0)
**Time from disease onset**	
Clinic visit, days	5 [1, 15]
Blood test, days	10 [5, 30]
Yamaguchi's criteria satisfaction, days	21 [9, 42]
Treatment initiation, days	35 [20, 68] (n = 87)
**Initial symptoms and findings**	
Fever, n (%)	45 (50.6)
Rash, n (%)	33 (37.1)
Arthralgia, n (%)	33 (37.1)
Sore throat, n (%)	27 (30.3)
Lymphadenopathy or splenomegaly, n (%)	3 (3.4)
Liver dysfunction, n (%)	2 (2.2)
**Incidence of Yamaguchi's criteria**	
Fever, n (%)	88 (98.9)
Rash, n (%)	80 (89.9)
Arthralgia / arthritis, n (%)	79 (88.8)
Sore throat, n (%)	60 (67.4)
Lymphadenopathy and/or splenomegaly, n (%)	67 (75.3)
Leukocytosis, n (%)	73 (82.0)
Liver dysfunction, n (%)	76 (85.4)
Negative rheumatoid factor and anti-nuclear antibody, n (%)	57/88 (64.8)
**Laboratory data before treatment initiation**	
White blood cell count, maximum, $\times 10^3/\mu L$	15973 (6330)
Neutrophil count, maximum, $\times 10^3/\mu L$	14062 (5794) (n = 78)
Hemoglobin, minimum, g/dL	10.4 (1.6) (n = 85)

	All (n = 89)
Platelet count, maximum, $\times 10^4/\mu L$	36.6 (15.4) (n = 86)
Aspartate aminotransferase, maximum, IU/L	75 [49, 135]
Alanine aminotransferase, maximum, IU/L	65 [38, 126] (n = 86)
Lactate dehydrogenase, maximum, U/L	495 [367, 773] (n= 85)
C-reactive protein, maximum, mg/dL	15.8 (8.7) (n = 88)
Erythrocyte sedimentation rate, mm/hr	91.5 (31.2) (n = 65)
Ferritin, ng/mL	4412 [1438, 8735] (n = 87)
Soluble IL-2 receptor, maximum, U/mL	1289 [845,1639] (n = 60)
Matrix metalloproteinase-3, maximum, ng/mL	91 [52, 188] (n = 59)

Data are n (%), mean (SD), and median [Q1-Q3].
‡ Tacrolimus, cyclosporine or methotrexate

Supplementary Table S2. Treatment and Outcomes

	All (n=89)
**Initial treatment**	
Glucocorticoid, n (%)	85/87 (97.7)
Glucocorticoid dose, mg/day	43.5 (19.1) (n = 85)
Glucocorticoid pulse therapy	9 (10.1)
**Immunosuppressive therapy within 90 days**	50/87 (57.5)
Biological agents, n (%)	29/87 (33.3)
Tocilizumab, n (%)	26/87 (29.9)
Infliximab, n (%)	1/87 (1.1)
Adalimumab, n (%)	1/87 (1.1)
Etanercept, n (%)	1/87 (1.1)
Other immunosuppressants, n (%) ‡	31/87 (35.6)
Cyclophosphamide, n (%)	1/87 (1.1)
Methotrexate, n (%)	15/87 (17.2)
Cyclosporin A, n (%)	18/87 (20.7)
Tacrolimus, n (%)	4/ 87 (4.6)
Mizoribine, n (%)	1/87 (1.1)
**Inadequate response to initial treatment**	
Glucocorticoid increase within 90 days from the initial therapy, n (%)	40/87 (46.0)
Day of treatment intensification from initial treatment, days	14 [8, 21] (n = 40)
**Macrophage activation syndrome**	
Macrophage activation syndrome, n (%)	19 (21.3)
Timing of macrophage activation syndrome	
Before initial treatment, n (%)	9/19 (47.4)
After initial treatment, n (%)	10/19 (52.6)
Days from initial treatment to macrophage activation syndrome, days	29 [16, 163] (n = 10)
**Observation period from initial treatment, years**	7.8 [3.6, 10.6] (n = 87)

	All<br>(n=89)
**Recurrence**	
Recurrence, n (%)	40 (44.9)
Day of first recurrence from initial treatment, years	1.4 [0.6, 2.9]<br>(n = 39*)
**Glucocorticoid discontinuation**	
Glucocorticoid discontinuation, n (%)	53/85 (62.4)
Day of glucocorticoid discontinuation, years	2.5 [1.3, 4.7]<br>(n = 53)

Data are n (%), mean (SD), and median [Q1-Q3].
* One patient experienced recurrence was not included because of no-treatment at initial disease onset.

Supplementary Table S3. Comparison of demographics and disease characteristics at diagnosis based on initial symptoms adjusted by their number

	Fever (n = 29.2)	Rash (n = 23.8)	Arthralgia (n = 19.0)	Sore throat (n = 15.0)	p
**Patients' characteristics**					
Age, years	41.8 (19.2)	47.9 (17.5)	48.7 (18.2)	51.2 (17.7)	0.257
Female, n (%)	19.8 (67.7)	21.3 (89.2)	13.3 (69.7)	9.8 (65.0)	0.141
**Time from disease onset**					
Clinic visit, days	1.3 [0.0, 7.0]	11.2 [3.0, 32.1]	7.2 [2.0, 31.8]	3.0 [0.0, 7.7]	0.003*
Blood test, days	7.0 [3.0, 12.3]	21.0 [13.1, 35.5]	9.5 [4.8, 31.8]	7.3 [3.0, 15.0]	<0.001*
Yamaguchi's criteria satisfaction, days	12.0 [6.8, 28.6]	31.2 [16.2, 47.4]	29.0 [12.4, 76.1]	14.0 [6.0, 24.0]	0.001*
Treatment initiation, days	30.0 [14.8, 57.0]	39.0 [25.0, 64.3]	46.0 [22.7, 127.3]	29.2 [18.0, 43.7]	0.081
**Initial symptoms**					
Fever, n (%)	29.2 (100)	3.2 (13.3)	6.8 (36.0)	5.8 (38.9)	<0.001*
Rash, n (%)	3.2 (10.9)	23.8 (100)	3.5 (18.4)	2.5 (16.7)	<0.001*
Arthralgia, n (%)	6.8 (23.4)	3.5 (14.7)	19.0 (100)	3.7 (24.4)	<0.001*
Sore throat, n (%)	5.8 (20.0)	2.5 (10.5)	3.7 (19.3)	15.0 (100)	<0.001*
Lymphadenopathy or splenomegaly, n (%)	0.5 (1.7)	0	0.5 (2.6)	0	0.495
Liver dysfunction, n (%)	1.5 (5.1)	0	0.5 (2.6)	0	0.268
**Incidence of Yamaguchi's criteria**					
Fever	29.2 (100)	23.5 (98.6)	18.7 (98.3)	14.7 (97.8)	0.659
Rash	26.3 (90.3)	23.8 (100)	14.2 (74.6)	13.7 (91.1)	0.008*
Arthralgia / arthritis	24.7 (84.6)	21.8 (91.6)	19.0 (100)	11.5 (76.7)	0.079
Sore throat	17.7 (60.6)	17.8 (74.8)	7.5 (39.5)	15.0 (100)	<0.001*
Lymphadenopathy and/or splenomegaly	22.3 (76.6)	17.0 (71.3)	14.2 (74.6)	11.5 (76.7)	0.966
Leukocytosis	24.4 (83.7)	21.3 (89.2)	13.9 (73.3)	11.4 (76.1)	0.430

	Fever (n = 29.2)	Rash (n = 23.8)	Arthralgia (n = 19.0)	Sore throat (n = 15.0)	p
Liver dysfunction	26.8 (92.0)	18.3 (76.9)	16.3 (86.0)	13.5 (90.0)	0.166
Negative rheumatoid factor and anti-nuclear antibody	18.8 (64.6)	14.2 (62.0) (n= 22.8)	11.7 (61.4)	11.3 (75.6)	0.760
**Laboratory data**					
White blood cell count, maximum, $\times 10^3/\mu L$	16339 (7087)	16986 (6139)	15178 (6106)	15069 (5633)	0.617
Neutrophil count, maximum, $\times 10^3/\mu L$	14462 (5362)	15079 (6334)	13161 (5966)	13435 (5805)	0.681
Hemoglobin, minimum, g/dL	10.3 (1.6)	10.5 (1.4)	10.2 (1.7)	10.5 (1.7)	0.889
Platelet count, maximum, $\times 10^4/\mu L$	35.0 (13.3)	34.4 (12.4)	43.2 (19.8)	34.7 (16.4)	0.258
Aspartate aminotransferase, maximum, IU/L	75.3 [52.7, 138.9]	74.2 [33.9, 102.1]	58.5 [47.5, 115.0]	88.8 [50.5, 164.8]	0.251
Alanine aminotransferase, maximum, IU/L	70.0 [43.0, 180.0]	59.5 [30.7, 86.1]	70.0 [29.0, 125.7]	106.0 [48.0, 126.0]	0.246
Lactate dehydrogenase, maximum, U/L	577 [373, 872]	464 [357, 739]	411 [237, 550]	566 [438, 986]	0.021*
C-reactive protein, maximum, mg/dL	17.9 (8.1)	15.3 (9.7)	15.7 (8.3)	14.4 (8.0)	0.376
Erythrocyte sedimentation rate, mm/hr	98.6 (28.2)	84.1 (30.9)	100.8 (20.1)	78.8 (38.5)	0.088
Ferritin, ng/mL	4926 [1483, 8529]	2636 [1361, 8588]	1916 [1066, 4577]	8430 [3350, 30284]	0.007*
Soluble IL-2 receptor, maximum, U/mL	1333 [821, 1601]	1154 [688, 1367]	1236 [816, 1441]	1729 [1363, 2406]	0.013*
Matrix metalloproteinase-3, maximum, ng/mL	78.8 [39.7, 270.2]	76.3 [44.7, 138.3]	154.2 [83.9, 418.7]	80.8 [51.8, 118.9]	0.050*

Data are n (%), mean (SD), and median [Q1-Q3]

Supplementary Table S4. Comparison of Treatment and Outcomes based on Initial Symptoms Adjusted by their Number

	Fever (n = 29.2)	Rash (n = 23.8)	Arthralgia (n = 19.0)	Sore throat (n = 15.0)	p
**Initial treatment**					
Glucocorticoid, n (%)	27.7 (98.2)	22.5 (98.5)	18.2 (95.6)	14.7 (97.8)	0.769
Glucocorticoid dose, mg/day	43.9 (18.5)	44.3 (22.6)	39.9 (14.6)	44.4 (20.5)	0.711
Glucocorticoid pulse therapy	2.3 (8.0)	2.3 (9.8)	0.8 (4.4)	3.5 (23.3)	0.143
**Immunosuppressive therapy within 90 days**					
Biological agents, n (%)					
Tocilizumab, n (%)	9.1 (32.2) (n =28.2)	7.4 (32.5) (n =22.8)	4.4 (23.3)	4.1 (27.2)	0.853
Infliximab, n (%)	0 (n = 28.2)	0 (n = 22.8)	1.0 (5.3)	0	0.081
Adalimumab, n (%)	0 (n = 28.2)	0.3 (1.5) (n = 22.8)	0.3 (1.8)	0.3 (2.2)	0.671
Etanercept, n (%)	0.5 (1.8) (n = 28.2)	0 (n = 22.8)	0.5 (2.6)	0	0.507
Other immunosuppressants, n (%) ‡					
Cyclophosphamide, n (%)	0.5 (1.8) (n = 28.2)	0 (n = 22.8)	0	0.5 (3.3)	0.390
Methotrexate, n (%)	4.3 (15.4) (n =28.2)	4.3 (19.0) (n = 22.8)	3.2 (16.7)	2.2 (14.4)	0.965
Cyclosporin A, n (%)	8.8 (31.4) (n = 28.2)	2.8 (12.4) (n = 22.8)	1.8 (9.6)	4.5 (30.0)	0.081
Tacrolimus, n (%)	2.0 (7.1) (n =28.2)	1.0 (4.4) (n = 22.8)	1.0 (5.3)	0	0.756
Mizoribine, n (%)	0 (n =28.2)	0.3 (1.5) (n = 22.8)	0.3 (1.8)	0.3 (2.2)	0.671

	Fever (n = 29.2)	Rash (n = 23.8)	Arthralgia (n = 19.0)	Sore throat (n = 15.0)	p
**Inadequate response to initial treatment**					
Glucocorticoid increase within 90 days from initial therapy, n (%)	14.6 (51.8) (n =28.2)	11.1 (48.5) (n =22.8)	5.6 (29.4)	7.8 (51.7)	0.333
Day of treatment intensification from initial treatment, days	15.8 [10.0, 22.6]	8.0 [5.5, 11.0]	15.2 [5.0, 20.2]	14.8 [10.2, 40.5]	0.044*
**Macrophage activation syndrome**					
Macrophage activation syndrome, n (%)	7.4 (25.4)	4.1 (17.1)	1.4 (7.5)	5.1 (33.9)	0.107
Timing of macrophage activation syndrome					
Before initial treatment, n (%)	3.3 (11.4)	2.3 (0.8)	0.8 (4.4)	2.5 (16.7)	0.551
After initial treatment, n (%)	4.1 (14.0)	1.8 (7.3)	0.6 (3.1)	2.6 (17.2)	0.266
Days from initial treatment to macrophage activation syndrome, days	17.9 [13.2, 25.6]	32.6 [14.7, 354.4]	56.1 [7.0, 228.1]	116.7 [14.9, 312.9]	0.216
**Observation period from initial treatment, years**	7.6 [4.1, 10.3]	8.5 [4.0, 10.9]	8.4 [3.4, 10.8]	7.1 [3.3, 10.2]	0.881

Data are n (%), mean (SD), and median [Q1-Q3]
